# Genetic basis of maize kernel protein content revealed by high-density bin mapping using recombinant inbred lines

**DOI:** 10.3389/fpls.2022.1045854

**Published:** 2022-12-15

**Authors:** Xin Lu, Zhiqiang Zhou, Yunhe Wang, Ruiqi Wang, Zhuanfang Hao, Mingshun Li, Degui Zhang, Hongjun Yong, Jienan Han, Zhenhua Wang, Jianfeng Weng, Yu Zhou, Xinhai Li

**Affiliations:** ^1^ Institute of Crop Science, Chinese Academy of Agricultural Sciences, Beijing, China; ^2^ College of Agriculture, Northeast Agricultural University, Harbin, Heilongjiang, China

**Keywords:** maize, protein content, genotyping by sequencing, high resolution linkage map, quantitative trait loci

## Abstract

Maize with a high kernel protein content (PC) is desirable for human food and livestock fodder. However, improvements in its PC have been hampered by a lack of desirable molecular markers. To identify quantitative trait loci (QTL) and candidate genes for kernel PC, we employed a genotyping-by-sequencing strategy to construct a high-resolution linkage map with 6,433 bin markers for 275 recombinant inbred lines (RILs) derived from a high-PC female Ji846 and low-PC male Ye3189. The total genetic distance covered by the linkage map was 2180.93 cM, and the average distance between adjacent markers was 0.32 cM, with a physical distance of approximately 0.37 Mb. Using this linkage map, 11 QTLs affecting kernel PC were identified, including *qPC7* and *qPC2-2*, which were identified in at least two environments. For the *qPC2-2* locus, a marker named IndelPC2-2 was developed with closely linked polymorphisms in both parents, and when tested in 30 high and 30 low PC inbred lines, it showed significant differences (*P* = 1.9E-03). To identify the candidate genes for this locus, transcriptome sequencing data and PC best linear unbiased estimates (BLUE) for 348 inbred lines were combined, and the expression levels of the four genes were correlated with PC. Among the four genes, *Zm00001d002625*, which encodes an S-adenosyl-L-methionine-dependent methyltransferase superfamily protein, showed significantly different expression levels between two RIL parents in the endosperm and is speculated to be a potential candidate gene for *qPC2-2*. This study will contribute to further research on the mechanisms underlying the regulation of maize PC, while also providing a genetic basis for marker-assisted selection in the future.

## 1 Introduction

Maize (*Zea mays* L.) is an important food and forage crop (Yang et al., 2013) that is cultivated globally across 20.2 million ha, and, in 2020, 116.2 million tons were produced (https://www.fao.org). Maize kernels are composed of approximately 10% protein, 72% starch, and 4% fat (Peña-Rosas et al., 2014). Chickens and pigs fed approximately 60% maize diet required an average of 15.5% and 16.5% protein during their growth, respectively (http://www.chinafeeddata.org.cn). This indicates that the protein content (PC) of maize was not sufficient to cater to the protein requirements of the feed. Therefore, utilizing the natural genetic variation controlling kernel PC in maize and introducing elite genome fragments into breeding are essential to satisfying feed demands and sustainable agriculture development.

Advances in next-generation sequencing (NGS) and biotechnology platforms have been vital for plant improvements and helped plant breeders to achieve greater genetic gains (Mutalik Desai et al., 2020). NGS can be divided into two strategies: whole genome resequencing (WGR) and reduced-expression sequencing (RRS) (Scheben et al., 2017). Per sample, the cost of RRS is lower than that of WGR but WGR is not affected to the same extent by the biases that impact RRS (Davey et al., 2011). Reads from genotyping-by-sequencing (GBS), one of the most widely used RRS methods, can be amplified using restriction enzymes, ligation, and polymerase chain reaction (PCR) prior to alignment with the reference genome (Poland and Rife, 2012; He et al., 2014). This process has been improved by the barcode system, which allows for discovery using genome-wide single-nucleotide polymorphisms (SNPs) with a lower error rate and cost (Elshire et al., 2011). SNP markers can be used to standardize and scale genotyping, and are known for their flexibility and abundance in the genome (Hoskins et al., 2001; Mammadov et al., 2012). The resolution of quantitative trait locus (QTL) mapping was previously limited as early genetic maps were mainly based on low-density markers, such as simple sequence repeat (SSR) markers, random amplified polymorphic DNA, and random fragment length polymorphisms (Mathivathana et al., 2019). Enough markers were produced by the reduced representation libraries of the genome and used for highly dense and accurate genetic linkage map construction (Gonda et al., 2019). GBS has been widely used in genetic studies as an efficient tool for an increasing number of plant species, including maize (*Zea mays* L.), barley (*Hordeum vulgare* L.), wheat (*Triticum aestivum* L.), rice (*Oryza sativa* L.), sorghum [*Sorghum bicolor* (L.) Moench], soybean [*Glycine max* (L.) Merr.], and cassava (*Manihot esculenta* Crantz) (Spindel et al., 2013; Glaubitz et al., 2014; Rowan et al., 2015; Zhou et al., 2016; Su et al., 2017; Wang et al., 2020).

The combination of multi-layered genetic analysis will assist in the prediction of candidate genes to study complex quantitative traits (Tang et al., 2021; Zhang et al., 2022). Seventy-four loci were found to be significantly associated with kernel oil concentration and fatty acid composition (*P*< 1.8 × 10^−6^) using expression (eQTL) mapping, linkage mapping, and co-expression analysis. Five candidate genes associated with oil metabolism (*LACS*, *WRI1a*, *ACP*, *FAD2*, and *COPII*) were chosen to investigate potential functional polymorphisms capable of causing phenotypic changes (Li et al., 2013). In recent years, a study that combined GWAS, linkage mapping, and eQTL analysis uncovered *GRMZM2G015534* and *GRMZM2G143008* as being involved in amino acid biosynthesis in maize (Deng et al., 2017). Genetic analysis of maize quality traits using multi-omics can greatly reduce the required time and labor when compared to fine mapping; however, few studies have investigated the genetic basis of PC in maize kernels using combined multi-omics.

To date, subsistent genes corresponding to maize storage proteins have been cloned, including *opaque1* (*o1*), *o2*, *o5*, *o7*, *floury1* (*fl1*), *fl2*, *fl4*, *mucronate* (*Mc*), and *defective endosperm B30* (*De-B30*) (Schmidt et al., 1990; Coleman et al., 1997; Kim et al., 2004; Kim et al., 2006; Holding et al., 2007; Myers et al., 2011; Wang et al., 2011; Wang et al., 2012; Wang et al., 2014). Nevertheless, the application of these genes in breeding is limited because of their defective endosperms. Kernel PC in maize is controlled by multiple genes with small individual and additive effects (Dudley et al., 1977), and analysis of the genetic basis of kernel PC will help to identify more favorable alleles for maize genetic improvements in the future. Genes/loci controlling PC traits can be analyzed using QTL analysis (Miao et al., 2020; Somegowda et al., 2022). Although some QTLs were detected at adjacent chromosome locations, genetic background, environmental factors, and their interactions have also been observed to have an effect in previous studies (Zhang et al., 2008; Li et al., 2009; Yang et al., 2014). Consequently, there are contrasting reports on QTL number, genetic effects, and distribution (Yang et al., 2016). Molecular marker development and the implementation of fine gene mapping to genetically improve traits could also not be achieved due to the absence of stable markers (Lozada et al., 2018; Ren et al., 2019; Ma et al., 2020; Lozada et al., 2021; Yepuri et al., 2022).

In the present study, we have constructed a recombinant inbred line (RIL) population using 275 lines derived from two elite inbred lines Ji846 and Ye3189 for the following purposes: (1) to construct a high-density linkage map based on bin markers; (2) to map QTLs for PC in maize kernels by combing phenotypes in RILs across three environments; (3) to develop PCR-based markers for major QTLs; and (4) to predict a candidate gene for a detected QTL.

## 2 Materials and methods

### 2.1 Plant materials and phenotypic evaluations

The RIL population consisted of 275 individuals and was derived from a cross between Ji846 (female) and Ye3189 (male), which are grown in the northeast spring maize area of China and are from the Lancaster and Reid heterotic groups, respectively. Compared with Ye3189, Ji846 had higher plant and ear height, more row kernel numbers, and higher resistance to maize head smut, however, it showed had lower 100-grain weight and single ear weight. The Ji846 and Ye3189 kernel type is biased towards flint and semi-dent, respectively. Phenotypic performance was evaluated in 2018 and 2019 at Gongzhuling (124.82°N, 43.50°E) and in 2019 at Harbin (126.68°N, 45.72°E); these locations are in the spring maize-growing region of China where these lines are commonly grown. A random incomplete block design was adopted for all experiments, with one-row plots, three replicates at each location, 17 plants in each row, 25 cm plant spacing, 60 cm row spacing, and a final density of 60,000 plants/ha. During the harvest period, five self-pollinating ears were collected from the center of each plot, and the samples were taken according to the standard procedure of phenotypic identification after air-drying. The kernels in the middle of the ear were mixed equally for each replication. The maize kernel PC was determined using an MPA Fourier near-infrared reflectance spectrometer (BRUKER, Germany) with a spectrum range of 4,000–12,000 cm^-1^, a scanning frequency of 64, and a resolution of 8 cm^-1^. The performance of each plot was represented by the average values for the different traits from three replicates.

### 2.2 Statistical analysis of phenotypic data

The raw phenotypic data were corrected using the ‘*lme*’ function in the R package ‘*lme4*’ with BLUE and the formula: Pheno ~1 + Line + (1|Rep) + (1|Env) + (1|Line : Env), where Pheno are phenotypic data representing traits. Line, Rep, and Env indicate the phenotypic data of inbred lines, the repetition in each environment, and environment, respectively. Other factors are regarded as random effects and a line that is fixed effects. Model matrix and grouping factors are separated by ‘|’, and the ‘:’ is used to represent the interactions between factors. Estimates for the phenotypic distributions, correlations, and parts of the QTL analysis were predominately based on BLUE.

According to Knapp et al. (1985), the modified formula: $H^{2}=\sigma_{G}^{2}/\left(\sigma_{G}^{2}+\sigma_{GY}^{2}/Y+\sigma_{GL}^{2}/L+\sigma_{GLY}^{2}/L\times Y+\sigma_{E}^{2}/L\times Y\times R\right)$ was employed to estimate the broad-sense heritability (*H^2^*) of traits across multiple environments, where $\sigma_{G}^{2}$ is the genotypic variance; the estimated interaction variance values for genotype × year, genotype × location, and genotype × location × year are represented by $\sigma_{GY}^{2}$ , $\sigma_{GL}^{2}$ , and $\sigma_{GLY}^{2}$ , respectively; *Y*, *L*, and *R* refer the number of years, locations, and replications per location, respectively; $\sigma_{E}^{2}$ is the error variance; all variances were estimated using the ‘*ASReml*’ R package.

### 2.3 DNA isolation, sequencing, and SNP identification

Leaf tissues were collected during the tasseling vegetative stage and freeze-dried at −80°C for genomic DNA isolation and extraction using the CTAB protocol (Murray and Thompson, 1980). Deep sequence for Ji846 and Ye3189 DNA from 275 RILs was processed according to the previously published GBS method (Zhou et al., 2016), in which *Nla*III (NEB) and *EcoR*I (NEB) were used for genomic DNA digestion with a TAA site that can be recognized by the restriction enzyme *Mse*I. Universal primers and index primers were employed to purify PCR products using Agencourt AMPure XP (Beckman). Fragments that were 350–400 bp (including adaptors and indexes) were isolated and purified for sequencing using the Illumina HiSeq2500 (Illumina, USA) platform. The raw sequence reads of these lines are public on NCBI (Accession: PRJNA627044).

To identify SNPs in the RIL population, Burrows-Wheeler Aligner (Li and Durbin, 2009) and GATK (McKenna et al., 2010) were used for alignment and SNP identification. The SNPs of the RILs were supported by a minimum of 1 base. ANNOVAR was employed to determine the physical position of each SNP based on the B73 RefGen_V4 sequence. Variant calling errors and the ratio of SNP alleles derived from Ji846 and Ye3189 were calculated using a sliding window approach. To ensure map quality, the few base types that did not appear in the parents were considered as abnormal bases; genotypes were screened to cover at least one individual marker in all progeny to ensure the integrity of genotypes; SNPs with segregation distortion of<0.001 were filtered out using Chi-square (χ^2^) tests, and those on the scaffold were filtered out to obtain high-quality SNP markers for subsequent map construction.

### 2.4 Bin map construction of the RIL population

A window size of 15 SNPs was used to scan the genotypic data with a step size of one SNP. If 11/15 of the sites in the window came from a single parent, they were considered homozygous, if not, they were considered heterozygous. Adjacent windows with the same genotypes were merged as a single block, whereas others were considered recombination breakpoints with variant genotypes. Bins were designated based on consecutive 100-kb intervals without any breakpoints across the entire population. The R/qtl function *est.map* (“kosambi” method) was manipulated to calculate the genetic distance between bin makers and linkage map construction.

### 2.5 QTL analysis for PC phenotypes in maize kernels

Phenotypic QTL locations in each of the three environments were determined using a composite interval mapping (CIM) method with the *R/qtl* software package (Broman et al., 2003), and BLUE was used to conduct a joint analysis across all environments. The threshold for defining the significant QTL was a logarithm of the odds (LOD) score of 3.0 calculated using 1,000 (*P<* 0.05) permutations in the analysis method. The genomic region wherein the LOD peak in the range of 1.5 decreased was considered the confidence interval of the QTL. The *fitqtl* function in the R ‘*qtl*’ package was used to calculate the proportion of phenotypic variation explained by each QTL. QTLs detected in different environments with overlapping confidence intervals or peak spacing within 20 Mb were considered to be single QTLs (Frascaroli et al., 2007).

### 2.6 Indel marker development

Genomic DNA was isolated and extracted using the CTAB protocol (Murray and Thompson, 1980). PCR was performed in a Bio-Rad T100™Thermal Cycler, with each reaction containing 10 mmolL^-1^ Tris-HCl, 0. 001% gelatin, 50 mmolL^-1^ KCl, 2.5 mmolL^-1^ MgCl_2_, 0.15 mmolL^-1^ dNTP, 10% glycerol, 0.25 μmolL^-1^ IndelPC2-2 primer (**Supplementary Table 1**), 0.5 µL Taq DNA polymerase, and 20 ng DNA template. Amplification conditions were 94°C for 5 min, followed by 35 cycles at 94°C for 1 min, 60°C for 2 min, and 70°C for 2 min.

Modified polyacrylamide gel electrophoresis (4.5%) was performed using a Bio-Rad sequencing plate device (38 cm × 30 cm × 0.4 mm). TEMED and APS were added to solidify the gel. Electrophoresis was performed for approximately 1 h at 50 W with a 2.5 μL PCR product sample in 1,800 mL 1 ×TBE buffer. The gel was soaked in 10% glacial acetic acid for 30 min, washed twice with water, 0.1% AgNO_3_ for 30 min, washed with water quickly once, and then soaked in 1.5% NaOH until the band type was distinguished. Sixty inbred lines with the highest and lowest PC in the inbred line population were selected to verify the availability of the markers. Significant differences between groups were analyzed using the *Signif* function in R software, and phenotype boxplots were drawn using the boxplot function in the *ggplot* R package.

### 2.7 Prediction of candidate genes

Correlations between the normalized expression level and phenotypic identification data were assessed in the maize inbred line population, which contained 348 lines. Normalized expression level data were obtained by RNA sequencing of maize kernels 20 days after pollination (20 DAP) using 150 bp paired-end Illumina sequencing, with an average of 7.7 Gb of high-quality raw sequencing data per line (**Supplementary Table 2**). Inbred lines were planted in Gongzhuling and Changping in 2018 and 2019 to collect the phenotypic data. Five ears in each block were self-pollinated, and 20 immature seeds from two ears in each block in Changping in 2019 were collected at 20 DAP for RNA-seq. The correlation coefficient and *P* value between gene expression levels and BLUE values for the phenotypes were calculated using the *Corrplot* package in the R software. The filtered working gene list of the maize genome was downloaded from MaizeGDB (http://www.maizegdb.org) to identify possible candidate genes within the QTL.

### 2.8 qRT-PCR analysis

*Zm00001d002625* expression was analyzed in inbred lines Ji846 and Ye3189. Total RNA was extracted 20 DAP from the endosperm using a FastPure^®^ Plant Total RNA Isolation Kit (Nanjing Vazyme Biotech Co. Ltd., China). Single-stranded cDNA was synthesized using the FastKing gDNA Dispelling RT SuperMix (Tiangen Biotech Co. Ltd., China). qRT-PCR was performed in Bio-Rad iQ5, with each reaction containing 100 ng of first-stand cDNA, 0.6 μL of 10 μmmolL^-1^ gene-specific primers, and 10 μL of SuperReal PreMix Plus (SYBR Green) (Tiangen Biotech Co. Ltd., China). Amplification conditions were 95°C for 15 min followed by 40 cycles at 95°C for 10 s and 58°C for 30 s. Relative expression of *Zm00001d002625* was calculated using the 2-ΔΔCq method (Livak and Schmittgen, 2001), and the variation in expression was estimated using three biological replicates. The maize *Tubulin* gene was used as an internal control to normalize the data. The primers used for real-time PCR and cDNA amplification are listed in **Supplementary Table 3**.

### 2.9 Sequence amplification of gene promoter and alignment analyses

Based on the results from QTL mapping, gene-specific primer pairs (**Supplementary Table 4**) were developed and used to amplify the promoter sequences of the candidate genes in Ji846 and Ye3189. PCR amplification was performed in 50 μL volumes containing 25 μL of 2×PCR buffer for KOD FX, 10 μL of 2mM dNTPs, 200 ng of genomic DNA, 1.5 μL of each forward and reverse primer (15 μmolL^−1^), and the suitable nuclease-free water in a T100™ Thermal Cycler (Bio-Rad Research, USA). The thermal cycling program was as follows: 95°C for 3 min, 34 cycles of 98°C for 30 s, 56°C for 30 s, and 68°C for 1 kb/min, and final extension at 68°C for 10 min. The amplified products were electrophoresed on a 1.0% agarose gel. The target bands were cut and purified using a Zymoclean™Gel DNA Recovery kit. Multiple DNA alignments were performed in DNAMAN. The transcription factor binding sites of candidate genes were predicted using a search of the PlantPAN3.0 (http://plantpan.itps.ncku.edu.tw/) database.

## 3 Results

### 3.1 Phenotype and genotype characteristics of the parental lines and RILs

**Figure 1:**
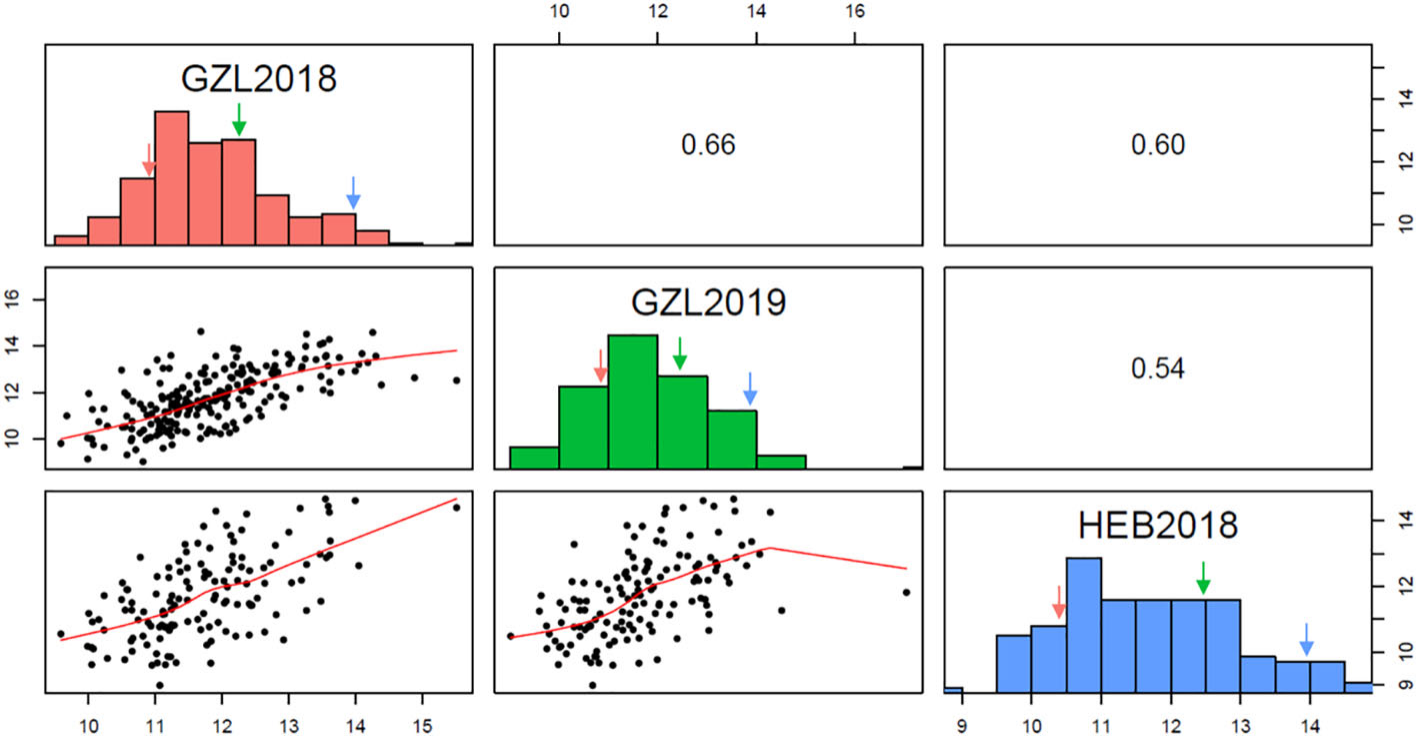
Frequency distribution of protein content (PC) and correlations across three environments. GZL2018, GZL2019, and HEB2018 indicate Gongzhuling in 2018, Gongzhuling in 2019, and Harbin in 2018, respectively. Red arrow: Ye3189; blue arrow: Ji846; blue arrow: mid-parent.

The RIL population and parental lines were evaluated at Gongzhuling in 2018 and 2019, and at Harbin in 2019(**Supplementary Table 5**). The PC of the female inbred line Ji846 was ~27.5% higher than that of the male Ye3189 based on the BLUE values, indicating a significant difference between the two parents (*P<* 0.05). The PC correlation coefficients ranged from 0.54 to 0.66 among the three environments. The mean of the Ji846 × Ye3189 RIL population was close to that of the mid-parent value, and the observations were distributed normally with transgressive segregation in all environments, indicating that the alleles responsible for increasing PC reside in both parents (**Figure 1**). ANOVA results showed that both genetic and environmental factors had significant effects on phenotypic variation. The broad-sense heritability(*h* ^2^) estimate of the kernel PC was high (81.10%), indicating that much of the phenotypic PC variation in the RIL population was genetically determined (**Table 1**).

**Table 1:** Combined analyses of variance for protein in Ji846 × Ye3189.

Source of Variation	Mean Square	SE
Genotype (G)	7.61E-01**	–
Location (L)	1.82E-08	–
Year (Y)	1.50E-08	–
L × G	1.21E-01**	–
Y × G	1.54E-02**	–
G × L × G	3.96E-01**	–
Block (B)	1.50E-08	–
Error (E)	1.48E-01**	–
*H^2^*	0.81	0.05

** indicate significance at P< 0.01. L × G, Y ×G, and G × L × G represent the genotype × location, genotype × year, and genotype × location × year interaction variances, respectively.

### 3.2 SNP detection in the RIL parental lines

**Figure 2:**
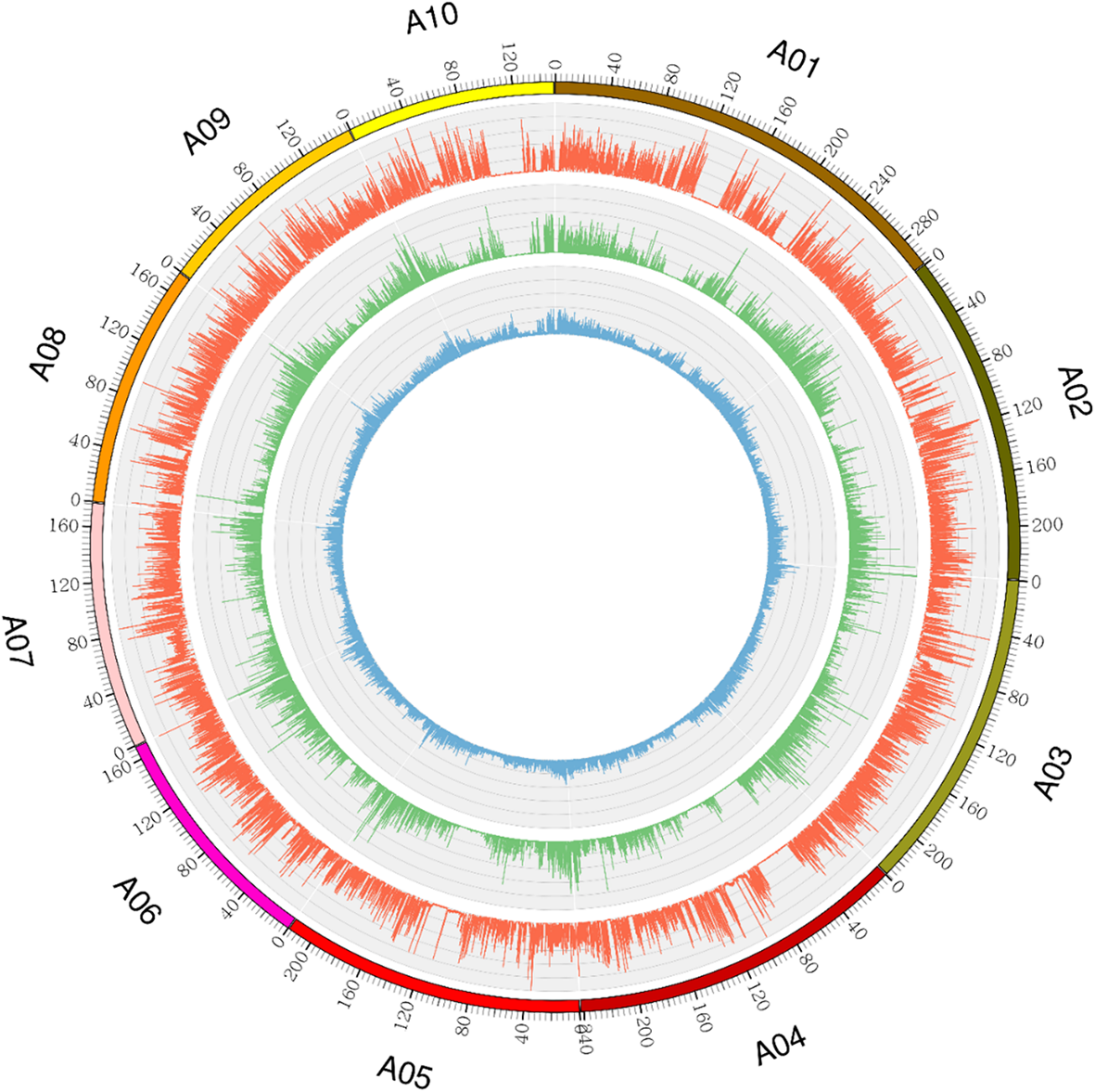
Genome-wide distribution of SNPs and genetic variants in the Ji846 and Ye3189 genomes. The outermost circle with scale represents the ten chromosomes. The orange histogram represents the density of SNPs that are polymorphic between Ji846 and Ye3189; the green histogram represents the density of polymorphic SNPs within the coding sequences of Ji846 and Ye3189; the blue histogram indicates the density of insertions and deletions (Indels) between Ji846 and Ye3189.

**Figure 3:**
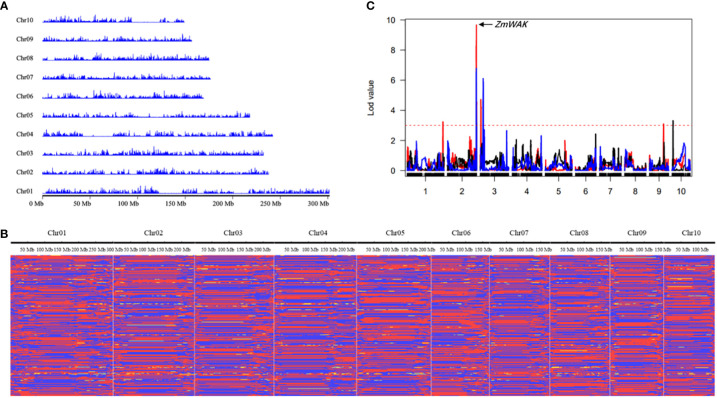
Genetic linkage map construction with bin markers. **(A)** SNP density distribution across the ten chromosomes. **(B)** Graphic genotype of 275 RILs. red, Ye3189 genotype; blue, Ji846 genotype; yellow, heterozygous genotypes. **(C)** Mapping of 2.09 and 3.04, which control maize head smut, in the RIL population. The curves with different colors represent the QTL regions according to the confidence intervals for linkage mapping under the three environments, respectively. Red, 2007 Harbin; black, 2008 Harbin; blue, 2009 Harbin; The x-axis indicates genetic positions across the maize genome in Mb. The y-axis indicates the LOD score of the detected QTL. The red dashed lines represent the LOD threshold.

Ji846 and Ye3189 were sequenced at 15.21× and 13.42× coverage depths, respectively. The results showed that 185,582,944 and 177,044,073 reads were mapped to the B73 RefGen_V4 genome with mapping rates of 98.09% and 98.31% for Ji846 and Ye3189, respectively. There were 11,132,691 and 10,275,040 SNPs identified and the heterozygosis rates were 41.75% and 33.51% in each population, respectively (**Figure 2**). A total of 18,192,527 SNPs were annotated by ANNOVAR, and approximately 87.80% were in the intergenic region, 5.25% in the introns, 1.90% in the exons, and 4.92% within 1 kb upstream or downstream of the genes (**Supplementary Table 6**). In total, 2,874,990 homozygous polymorphic SNPs were obtained between Ji846 and Ye3189 as *aa* × *bb* segregation patterns. Sequencing of the 275 RILs using the GBS strategy resulted in 1,447,346–3,741,336 mapping reads, with an average sequencing depth of 11.85, and mapping rates ranged from 95.83% to 98.03%. An average of 221,271 SNPs were obtained for each line, with a minimum of 105,512 and a maximum of 320,184 (**Figure 3A**).

### 3.3 Genetic linkage map construction and QTL mapping for kernel PC

After quality control and filtering, 77,680 SNPs, accounting for 2.70% of the total SNPs between Ji846 and Ye3189, were used to construct linkage maps. The sliding-window approach was applied to construct a bin map for 275 RILs. A total of 15,611 breakpoints were detected for 275 RILs with 56.7 breakpoints per individual (**Supplementary Figure 1**). Subsequently, there were 6,433 bin markers generated, and a high-density genetic map was constructed with a length of 2180.93 cM (**Supplementary Figures 2, 3**). The average distance ranged from 0.32 cM for chromosomes 2 and 3 to 0.37 cM for chromosomes 7 and 9, and the linkage interval between adjacent bins ranged from 2.32 cM to 4.64 cM, with an average of 3.31 cM (**Figure 3B**; **Table 2**). In addition, 77.79% of the bins were<300 kb in length, with 4.15% of the bins being longer than 1 Mb (**Supplementary Figure 4**). To evaluate the quality of the genetic map, bin markers were mapped to the maize B73 RefGen_V4 reference genome. The markers in the 10 linkage groups aligned well with the scatter plot of the B73 reference chromosome, indicating that there was appropriate collinearity between the maize B73 reference genome and identified bin markers (**Supplementary Figure 5**). To validate the power of this genetic map, QTLs for maize head smut, a trait previously mapped using SSR markers from this population, were mapped using the CIM function in R/qtl. QTL mapping revealed that 11.22% of the phenotypic variation could be explained by one major QTL located in a 231.15–244.44 Mb interval on chromosome 2. This locus included one cloned gene, *ZmWAK* (238,607,011–238,613,457), which confers quantitative resistance to maize head smut (**Figure 3C**) (Zuo et al., 2015). The results of this study narrowed the range of the QTL intervals, demonstrating the high resolution and accuracy of this bin map.

**Table 2:** Characteristics of the high-density genetic map derived from a cross between Ji846 and Ye3189.

Chr. ^a^	No. SNPs ^b^	No. markers ^c^	Physical distance (Mb)	Genetic distance (cM)	Avg. distance between markers (cM)	No.< 5 cM Gap	Max.gap (cM)
1	10,773	1031	301,476,924	360.88	0.35	1,031	3.19
2	9,213	812	237,917,468	258.14	0.32	811	2.55
3	10,214	729	232,245,527	233.27	0.32	728	3.95
4	8,665	759	242,062,272	247.66	0.33	758	2.58
5	6,335	612	217,959,525	204.27	0.33	611	4.64
6	7,218	548	169,407,836	183.40	0.34	547	2.49
7	6,923	538	176,826,311	197.27	0.37	537	4.36
8	7,949	567	175,377,492	192.74	0.34	566	4.14
9	5,402	467	157,038,028	171.88	0.37	466	2.32
10	4,988	370	149,632,204	131.41	0.36	369	2.91
Total	77,680	6433	2,059,943,587	2180.93	0.34	6,424	4.64

^a^Chr., indicates chromosome

^b^No. SNPs, the number of SNPs on a chromosome

^c^No. markers, the number of markers on a chromosome

**Figure 4:**
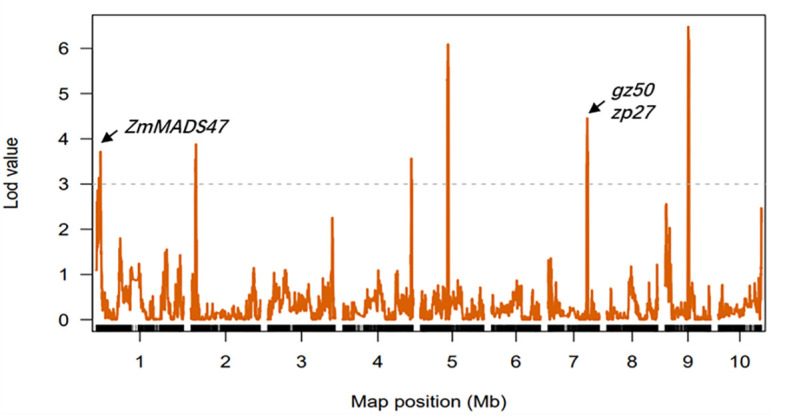
Mapping of QTLs for protein on the ten maize chromosomes in three different environments (combined). The curves indicate the physical positions (x-axis) of the bin markers against the LOD scores (y-axis) for QTLs detected on each of the ten chromosomes. The gray dashed lines represent the LOD threshold.

QTLs for kernel PC in each environment were detected based on a linkage map. Eleven QTLs were identified, two of which were detected in multiple environments and were distributed on chromosomes 2 and 7 (**Supplementary Figure 6**). The confidence intervals for these 11 QTLs spanned physical distances of 1.15–14.10 Mb, with an average of 5.14 Mb. The phenotypic variation explained by each QTL ranged from 4.21% to 14.56%, with a mean of 6.82%. In addition, six QTLs for kernel PC were detected from the analysis in the three environments when combined with the BLUE values (**Figure 4**). The confidence intervals for these six QTLs spanned physical distances of 3.40–14.10 Mb, with an average of 6.41 Mb. The phenotypic variation explained by each QTL ranged from 4.21% to 7.76%, with a mean of 5.53% (**Table 3**). The *qPC9* locus with a 14.56% contribution to phenotypic variation was only identified in the Gongzhuling environment in 2018, indicating that specific QTLs might be strongly expressed in a certain environment. *qPC7* and *qPC2-2* were identified in at least two environments as stable loci that could be used to identify candidate genes. *qPC7* (Chr7:116.1-137.7 Mb) and *qPC2-2* (Chr2:14.45-19.95 Mb) explained ~7.32% and ~6.01% of the phenotypic variation, respectively, and were detected in multiple environments and considered stable loci that could be used to identify candidate genes. The reported *gz50* and *zp27* genes, which encode 50-kDa and 27-kDa *γ*-zein genes, directly affected the PC of kernels and were located in *qPC7*, indicating the accuracy of the localization results in this study.

**Table 3:** QTLs detected for kernel protein content in the RILs.

Name^a^	Chr.^b^	Env.^c^	Marker interval ^d^	Interval (Mb) ^e^	PVE^f^	ADD^g^
*qPC1*	1	BLUE	mk7-mk89	4.65-18.75	4.21	0.31
*qPC2-1*	2	2019GZL	mk1057-mk1080	4.00-6.95	5.12	0.57
*qPC2-2*	2	2018GZL	mk1121-mk1137	14.85-18.40	6.64	0.54
*qPC2-2*	2	2018HEB	mk1120-mk1147	14.65-19.95	6.55	0.66
*qPC2-2*	2	BLUE	mk1119-mk1137	14.45-18.40	4.75	0.32
*qPC3-1*	3	2018GZL	mk1940-mk1959	19.85-25.15	4.31	-0.43
*qPC3-2*	3	2018HEB	mk2391-mk2406	194.15-198.55	12.43	0.91
*qPC4*	4	BLUE	mk3276-mk3301	234.55-237.95	4.73	-0.32
*qPC5-1*	5	2019GZL	mk3618-mk3637	89.70-96.45	5.27	0.58
*qPC5-1*	5	BLUE	mk3622-mk3638	91.65-97.25	6.24	0.38
*qPC5-2*	5	2018GZL	mk3686-mk3698	142.40-148.55	5.62	0.5
*qPC7*	7	2018HEB	mk4759-mk4776	116.10-120.70	9.13	-0.78
*qPC7*	7	2019GZL	mk4798-mk4816	125.15-128.40	7.35	-0.68
*qPC7*	7	BLUE	mk4828-mk4846	131.95-137.70	5.47	-0.35
*qPC9*	9	2018GZL	mk5780-mk5786	69.15-74.75	14.56	-0.80
*qPC9*	9	BLUE	mk5794-mk5802	76.90-82.55	7.76	-0.42
*qPC10*	10	2019GZL	Mk6428-mk6433	147.95-149.10	5.87	0.61

^a^Names of the QTLs on the individual chromosomes.

^b^Chr., chromosome.

^c^QTLs in a specific environment:2018HEB is Harbin in 2018; 2018GZL is Gongzhuling in 2018; 2019GZL is Gongzhuling in 2019; BLUE represents joint analysis.

^d^Marker interval, the markers flanking the QTL.

^e^Interval, confidence interval between two bin markers.

^f^PVE, phenotypic variance explained by an individual QTL.

^g^ADD, the value of additive genetic effect.

### 3.4 Marker development for qPC2-2

**Figure 5:**
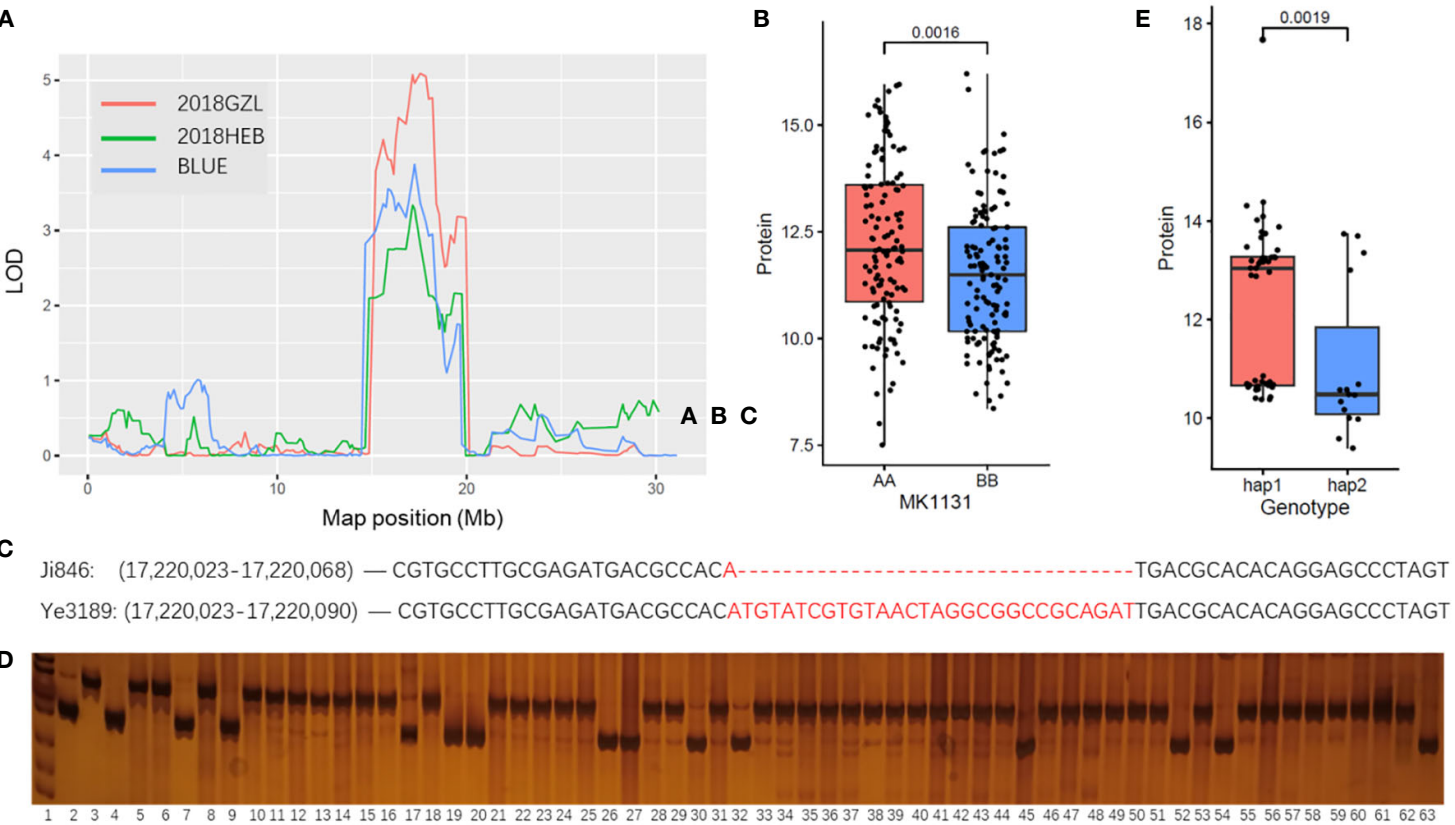
Distribution of the phenotypes and QTLs identified on chromosomes in the different environments assessed in this study. **(A)** The curves indicate the physical positions (x-axis) of bin markers against the LOD scores (y-axis) for QTLs detected on *qPC2-2*. Different colors represent different environments. 2018GZL and 2018HEB represent Gongzhuling and Harbin in 2018, respectively; BLUE represents joint analyses. **(B)** Tests for differences among phenotypic values for the BLUE values of PCs associated with MK1131 in maize inbred lines. AA represents the Ji846 genotype; BB represents the Ye3189 genotype. The y-axis indicates the phenotypic values for the BLUE values of PC. **(C)** Indels between Ji846 and Ye3189 around the peak position (MK1131) in *qPC2-2*. **(D)** Genotyping of maize inbred lines with 30 high and 30 low PCs using marker IndelPC2-2, lane 1: marker; lane 2: genotype of Ji846; lane 3: genotype of Ye3189; lane 4-63: genotypes of 60 inbred lines. **(E)** Tests for differences among phenotypic BLUE values of PCs associated with the IndelPC2-2 marker in maize inbred lines with 30 high and 30 low PCs. Hap1 represents Ye3189 genotype; hap2 represents Ji846 genotype. The y-axis indicates the phenotypic values for the BLUE values for PC.

To further verify the correlation between the *qPC2-2* locus and PC trait, an insertion and deletion (Indel) marker was developed based on the variation between biparental bases. The genetic linkage analysis results implied that the strongest signal associated with the phenotype was at the *qPC2-2* locus of MK1131 (Chr2:17.15 Mb) in two different environments (**Figure 5A**). SNPs between Ji846 and Ye3189 located in MK1131 were calculated and categorized according to their parental alleles. A total of 122 SNPs matched the Ji846 parental genotype and 129 matched the Ye3189 genotype. The phenotypic values for kernel PC in the Ji846 × Ye3189 RIL population differed significantly (*P* = 1.6E-03) (**Figure 5B**). We also developed a PCR-based marker, IndelPC2-2, with closely linked polymorphisms in both parents near MK1131 (**Figure 5C**), which was used to genotype 30 high and 30 low PC maize inbred lines (**Figure 5D**), and the phenotypes of the diverse genotypes were also significantly different (*P* = 1.9E-03) (**Figure 5E**). Therefore, IndelPC2-2 could be utilized to enhance breeding efficiency using marker-assisted selection (MAS) with the PC trait.

### 3.5 Candidate gene prediction

**Figure 6:**
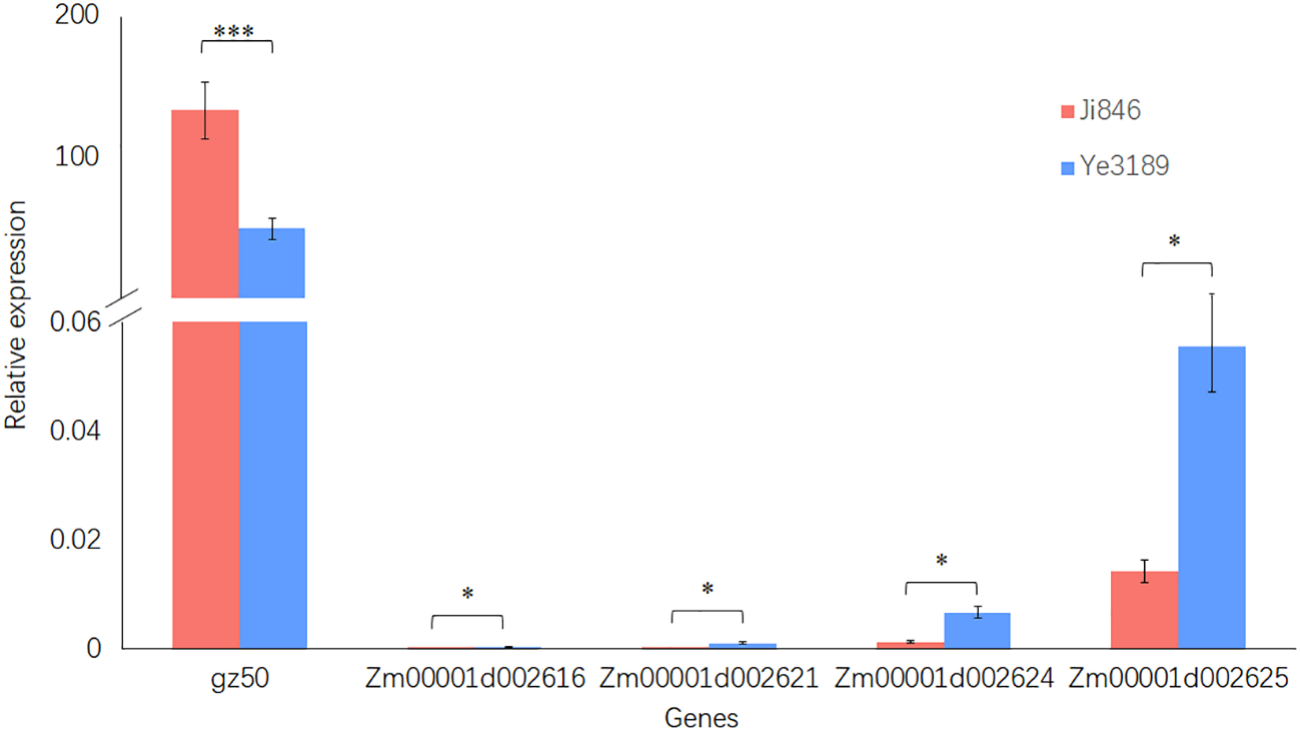
Relative quantitative expression analysis of *Zm00001d002625* in the endosperm (20 DAP) of Ji846 and Ye3189 using qRT-PCR. The red bars represent Ji846 (high-protein parent), and the blue bars represent Ye3189 (low-protein parent). The horizontal axis of the graph indicates the different genes, and the vertical axis indicates the relative expression levels. The data are presented as the means of three biological and technical replicates ± standard errors. * and *** indicate *P<* 0.05 and *P<* 0.001, respectively, by a t-test.

To rapidly identify candidate genes within the stable QTL locus *qPC2-2*, correlation analysis using RNA-seq was adapted in this study. *gz50* and *zp27* genes are reported to encode 50-kDa and 27-kDa *γ*-zein genes that directly affect kernel development in *qPC7*. Correlation analysis using RNA-seq data from 348 inbred lines showed that only the *gz50* gene expression level was significantly correlated with PC (*r* = 0.24, *P* = 6.21E-06), indicating the rationality of the adopted approach (**Supplementary Table 7**). For *qPC2-2*, the same method was adapted to predict the candidate genes in the interval Chr2:16.80 Mb to 17.25 Mb around peak marker MK1131. Four of the 12 gene expression levels were significantly correlated with the kernel PC BLUE value in the maize inbred line population (*r* = -0.11–0.22, *P<* 0.05). Furthermore, the qRT-PCR validated *gz50* for *qPC7*, and *Zm00001d002616*, *Zm00001d002621*, *Zm00001d002624*, and *Zm00001d002625* for *qPC2-2* showed significant differences in gene expression levels in the endosperms of Ji846 and Ye3189 (20 DAP). Notably, among the four genes for *qPC2-2*, the expression of *Zm00001d002625* (closest to MK1131) of Ye3189 was approximately 3.9 times than that in Ji846, which was the highest among the candidate genes (**Figure 6**). We obtained the sequence of promoter region for Zm00001d002625 gene in Ji846 and Ye3189. A 1 bp variation on 1250 bp upstream of the initiation codon in the promoter region of the *Zm00001d002625* gene resulted in the absent binding motif GATCT of GATA-type transcription factors in Ji846 (**Supplementary Figure 7**; **Supplementary Tables 8, 9**). According to the maize gene annotation database (maize GDB, www.maizegdb.org), there was 60% homology between *Zm00001d002625* and *At4G00750* in *Arabidopsis thaliana*, which encodes an S-adenosyl-L-methionine-dependent methyltransferase superfamily protein; except for *Zm00001d002625*, none of the other three genes encoded proteins with functional annotations. SAM serves as the methylation donor in transmethylation reactions, probably causing a genome-wide disruption in storage proteins in developing endosperms due to methylation changes (Yan et al., 2022). In summary, *Zm00001d002625* was speculated to be a potential candidate gene associated with kernel PC in *qPC2-2*.

## 4 Discussion

Improving the PC trait in maize kernels is one of the prime objectives of most breeding programs around the world. A limited number of the genes involved in starch metabolism are also known to have major effects on the PC in maize kernels based on the analysis of well-known maize mutants (Coleman et al., 1997; Kim et al., 2006; Wang et al., 2014). The identification of QTLs related to PC will thus aid in MAS breeding and the accumulation of elite alleles in natural variations using map-based cloning strategies (Raihan et al., 2016; Yang et al., 2016).

In general, segregation distortion, heterozygosity, allele switching, excessive single-cross events, and unexpected double recombinants, such as genotyping errors, occur during the construction of an SNP-based genetic linkage map (Cartwright et al., 2007). The abundance of missing data points and sequencing errors may cause an expansion of the genetic distance between markers in a genetic map or misplaced markers in the map due to the limited sequence depth (Spindel et al., 2013; Ma et al., 2020). In this study, the average effective depth of resequencing for the parents was approximately 14.32-fold; although 7,300,643 SNPs were obtained using the filtering steps, SNPS with abnormal bases, integrity, and partial separation were filtered out using stringent criteria. This resulted in 77,680 markers and samples with high-quality scores, which ensured the construction of a genetic map with a high level of accuracy (Huang et al., 2009; Jaganathan et al., 2015; Saxena et al., 2017; Mathivathana et al., 2019). The linkage map derived from the Ji846 × Ye3189 RIL population was constructed previously using 160 SSR markers and 49 AFLP markers, which covered 3,302.8 cM of the maize genome, and the average marker interval was 15.8 cM. In contrast to PCR-based SSR markers, a recombination breakpoint between the two bin markers with an average of approximately 0.34 cM can be identified using GBS, which represents a 46-fold improvement in the resolution of recombination breakpoints. Compared to the genetic linkage map constructed with general size populations for genetic dissection in the previous study, our genetic linkage map has a relatively higher resolution (Chen et al., 2014; Zhou et al., 2016; Su et al., 2017). It is worth mentioning that this population is suitable for the genetic dissection of maize quality traits due to the diversity of the kernel characteristics between parents. However, it is worth noting that many QTLs will likely be missed if only one bi-parental population is used due to the lack of polymorphism between the two parental lines. As a consequence, more elite alleles could be identified by using inbred populations and multiple parental populations. (Wang et al., 2018).

The selection of DNA-based markers, mapping functions, methods of phenotypic data collection, and mapping population type and size are all factors that affect QTL identification and analysis and the development of linkage maps (Hyne et al., 1995; Poland and Nelson, 2011; Su et al., 2016). Compared with the QTLs detected in the bi-parent population, the *qPC1*, *qPC2-1*, and *qPC3-1* loci with 4.21–5.12% contributions to phenotypic variation were consistent with previous results (Dudley et al., 2004; Zhang et al., 2008; Guo et al., 2013; Yang et al., 2014; Zhang et al., 2015). The *PC2-2*, *qPC3-2*, *qPC5-1*, *qPC5-2*, *qPC7*, and *qPC9* loci with 5.47−14.56% contributions to phenotypic variation were also similar to those identified in previous studies (Dudley et al., 2004; Zhang et al., 2008; Cook et al., 2012; Yang et al., 2014). The consistent and stable QTLs detected in diverse genetic backgrounds can be used for QTL fine-mapping and the identification of candidate genes. The previously unreported *qPC4* locus demonstrated that the QTLs had a population-specific effect (Dudley et al., 2004; Yang et al., 2014). The *qPC9* locus explained the largest phenotypic variation; nevertheless, it was detected only once in a sample from Gongzhuling in 2018. QTL × environment interaction is an important property of QTLs, even for highly heritable traits, such as plant height, and these interactions are trait- and gene-specific (Li et al., 2003; Mathivathana et al., 2019). In addition, it remains unclear whether inconsistent QTL detection is due to true differential trait expression across environments (Yan et al., 2003).

In our study, *qPC2-2* and *qPC7* were identified as environmentally stable loci, which indicates that their QTL-by-environment interaction had a smaller effect (Li et al., 2003). The interaction of QTL quantitative traits has been well established and indicates the potential of combining favorable alleles to improve plant performance (Lefebvre and Palloix, 1996; Thabuis et al., 2004; Lu et al., 2012; Chunthawodtiporn et al., 2019). Zeins, which are the main storage proteins in endosperm maize kernels, have evolved into four classes based on their chemico-physical properties: alpha (*α*), beta (*β*), gamma (*γ*), and delta (*δ*) (Moose et al., 2004; Xu and Messing, 2009; Wang et al., 2011; Xiang et al., 2018). *gz50* encodes a 50-kDa *γ*-zein gene that directly affects kernel PC and is located in *qPC7*. The expression of *gz50* is strictly regulated through the identification of conserved cis-elements in the promoter and corresponding transcription factors (TFs) (Zhang et al., 2019). ZmMADS47, a MADS box-containing TF in Chr1:17,963,695–17,987,258 at the *qPC1* locus, was speculated to affect the PC of kernels by binding to the CATGT motif in the promoter of *α*-zein and 50-kDa *γ*-zein (Qiao et al., 2016). The 5.98% phenotypic contribution rate of *qPC2-2* was lower than that of *qPC7*, and previous studies indicated that even QTLs with minor effects could contain genes that cause extreme changes in corresponding phenotypes (Ishii et al., 2013; Xing et al., 2015). Maize breeding is process involving pyramiding favorable alleles, and it is known that important genes or major QTLs for agronomic traits are selected and even potentially fixed in the process of maize domestication or early breeding (Doebley et al., 1997; Wang et al., 2005; Wang et al., 2018). Thus, *qPC2-2* could potentially be used as a QTL in commercial breeding. A PCR-based marker, IndelPC2-2, was also developed, which may be linked to candidate genes or as a distinct regulatory element that affects the expression of candidate genes. IndelPC2-2 can be used to enhance breeding efficiency using MAS with the PC trait.

The combination of multilayered genetic analysis will accelerate QTL mapping, gene cloning, and molecular breeding in crops. Transcriptome-wide association studies with respect to a trait and analyses of the expression levels of genes across the whole genome have been conducted to assist in the prediction of candidate genes when studying complex traits (Li et al., 2013; Deng et al., 2017; Tang et al., 2021; Zhang et al., 2022). To rapidly identify candidate genes within the stable QTL locus *qPC2-2*, correlation analysis using RNA-seq was adapted in the present study. *Zm00001d002625* is speculated to be a potential candidate gene for *qPC2-2.* The alteration of one base upstream of the start codon of parents resulted in the variation of C2H2-GATA family transcription factor binding element, However, there are few studies on the effect of C2H2-GATA family transcription factor on PC of maize kernels. In addition, the promoter region of the gene may not be limited to 1250 bp before ATG of the initiation codon, and there may be the presence of distal regulation. *Zm00001d002625* has 60% homology with *At4G00750* in *Arabidopsis thaliana*, which encodes an S-adenosyl-L-methionine-dependent methyltransferase superfamily protein. Previous studies have demonstrated that the *SHMT4* mutation caused a disruption in storage protein composition, probably through SAM production, resulting in genome-wide methylation changes in developing endosperms (Yan et al., 2022). *Zm00001d002625* may encode an S-adenosyl-L-methionine-dependent methyltransferase superfamily protein, S-adenosyl-L-methionine synthetase, which catalyzes the conversion of methionine to SAM and serves as the methylation donor in transmethylation reactions and an intermediate in polyamine and ethylene biosynthesis (Li et al., 2011).

In conclusion, herein, we constructed a high-density maize linkage map after the large-scale development of markers using GBS of an RIL population. These results indicate that this high-density map can be used for accurate and efficient QTL mapping. Using this map, we have mapped PC in maize kernels and identified stable QTLs in multiple environments. Furthermore, we have developed an Indel marker associated with PC at the *qPC2-2* locus, *Zm00001d002605*, which was speculated to be a potential candidate gene within the QTL. This study will contribute to further research into the mechanisms that control PC, while also providing a basis for MAS in molecular breeding to further improve high-value traits.

## Data availability statement

The datasets presented in this study can be found in online repositories on NCBI (Accession: PRJNA627044).

## Author contributions

XL and ZZ performed the experiments and wrote the paper. YW, RW, ZH, ML, DZ, HY and JH, ZW performed the experiments and revised the paper. JW, YZ and XHL designed the experiments. All authors contributed to the article and approved the submitted version.
