# Supplementary material for: Genetic basis of maize kernel protein content revealed by high-density bin mapping using recombinant inbred lines
Source: Front Plant Sci. 2022 Dec 15;13:1045854. doi: 10.3389/fpls.2022.1045854 (PMC9798238; doi:10.3389/fpls.2022.1045854)
Supplement: Supplementary file 1 [file DataSheet_1.docx]

Supplementary Material

**Genetic basis of maize kernel protein content revealed by high-density bin mapping using recombinant inbred lines**

**Authors**

Xin Lu ^1†^, Zhiqiang Zhou^1†^, Yu Zhou^2^, Yunhe Wang^1,2^, Ruiqi wang^1^, Zhuanfang Hao^1^, Mingshun Li^1^, Degui Zhang^1^, Hongjun Yong^1^, Jienan Han^1^, Zhenhua Wang^2^, Jianfeng Weng^1^, Yu Zhou^2^*, and Xinhai Li^1^*

**Affiliations**

^1^ Institute of Crop Science, Chinese Academy of Agricultural Sciences, Zhongguancun South Street, Haidian District, Beijing, 100081, China

^2^ College of Agriculture, Northeast Agricultural University, Mucai Street, XiangFang District, Harbin, Heilongjiang, 150030, China

*** Corresponding author**

Xinhai Li and Yu Zhou

E-mail: lixinhai@caas.cn; [zhouyu0924@126.com](mailto:zhouyu0924@126.com)

**†Equal contributors**

Lu Xin^1†^ and Zhiqiang Zhou ^1†^

E-mail: [18745797462@163.com](mailto:18745797462@163.com); [zzq880211@163.com](mailto:zzq880211@163.com).


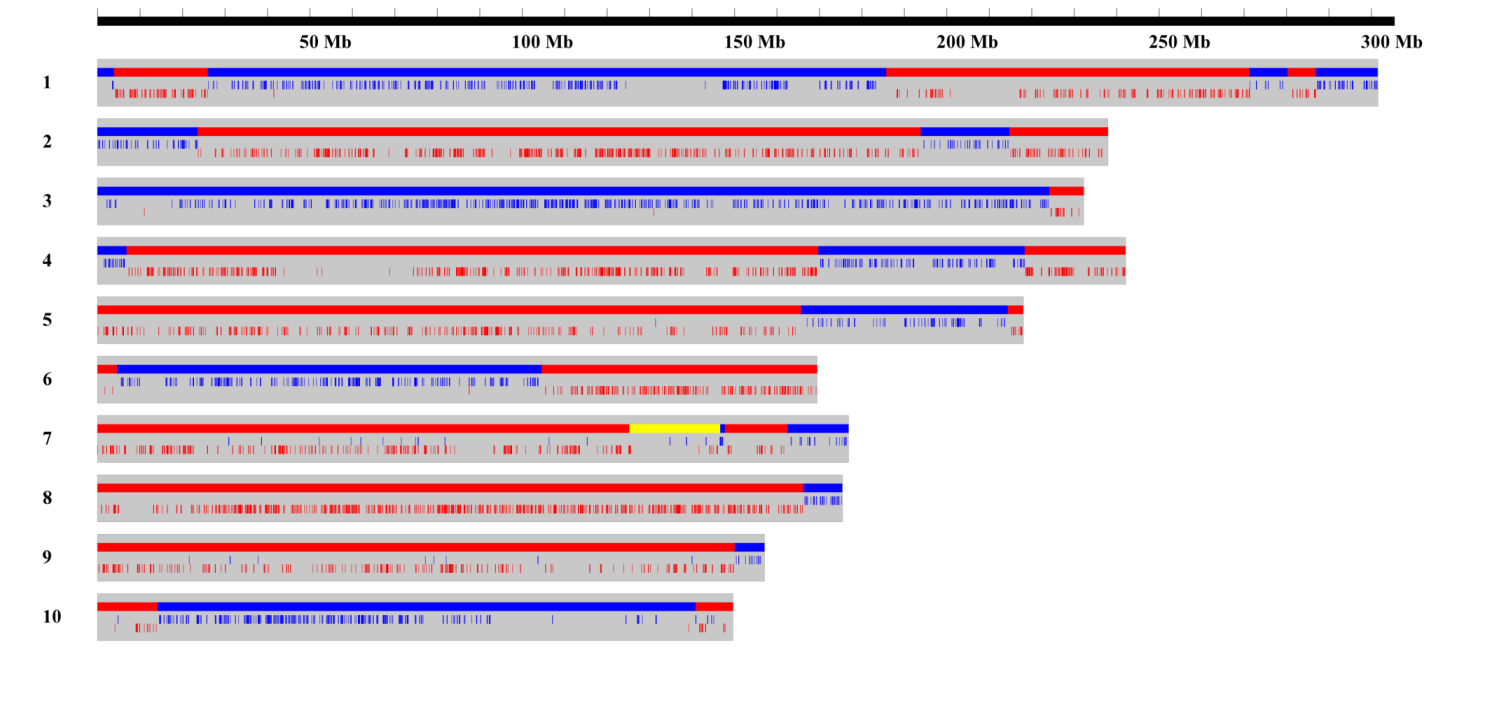


**Supplementary Figure 1.** **Recombination map of R011.** All positions are transformed to physical positions according to the B73 RefGen_V4 sequence. Red lines: homozygous Ye3189 genotype; Blue lines: homozygous Ji846 genotype; Yellow lines: the heterozygous region.

**
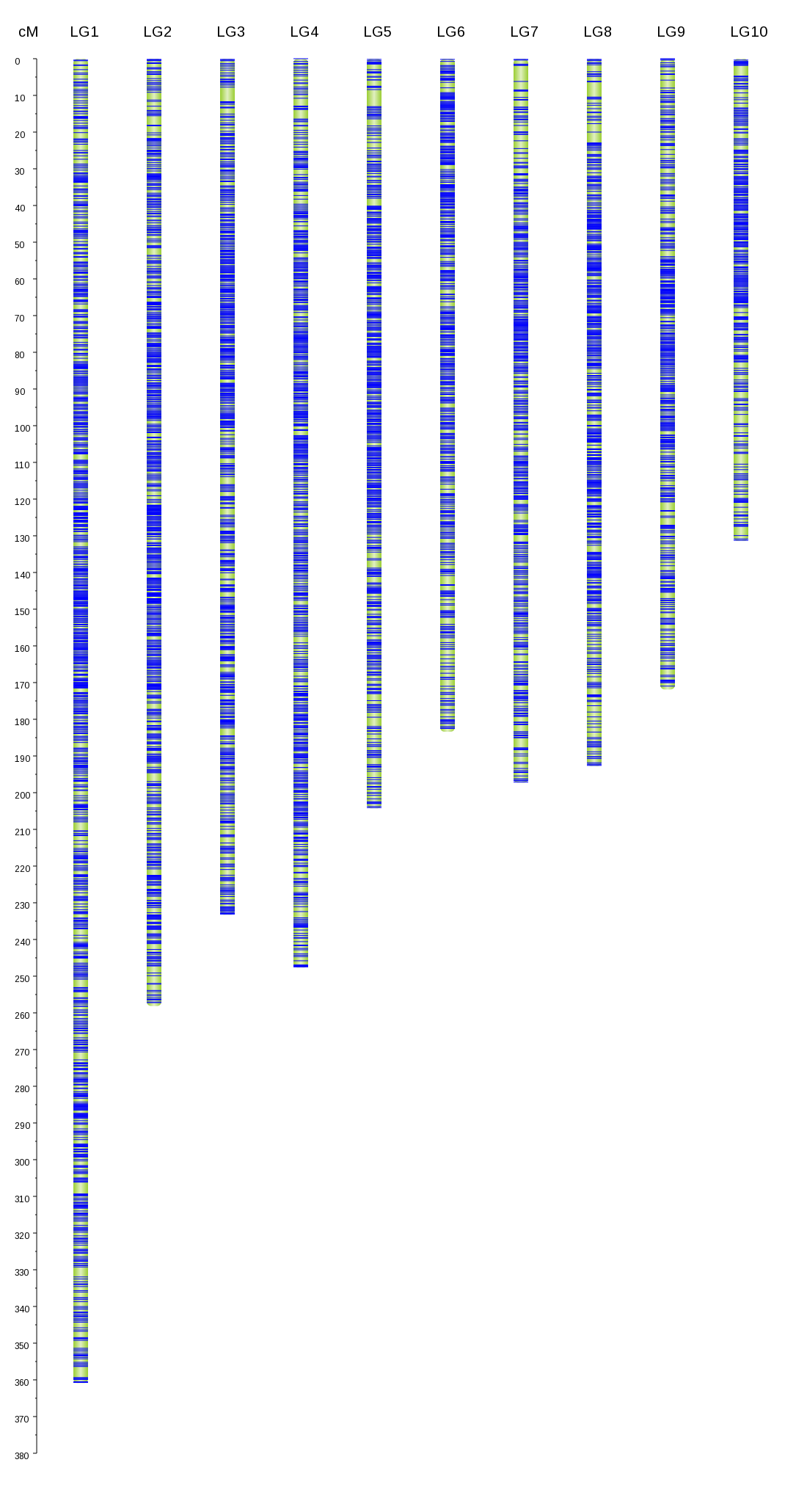
**

**Supplementary Figure 2.** **Distribution of the linkage group (LG) marker.**

**
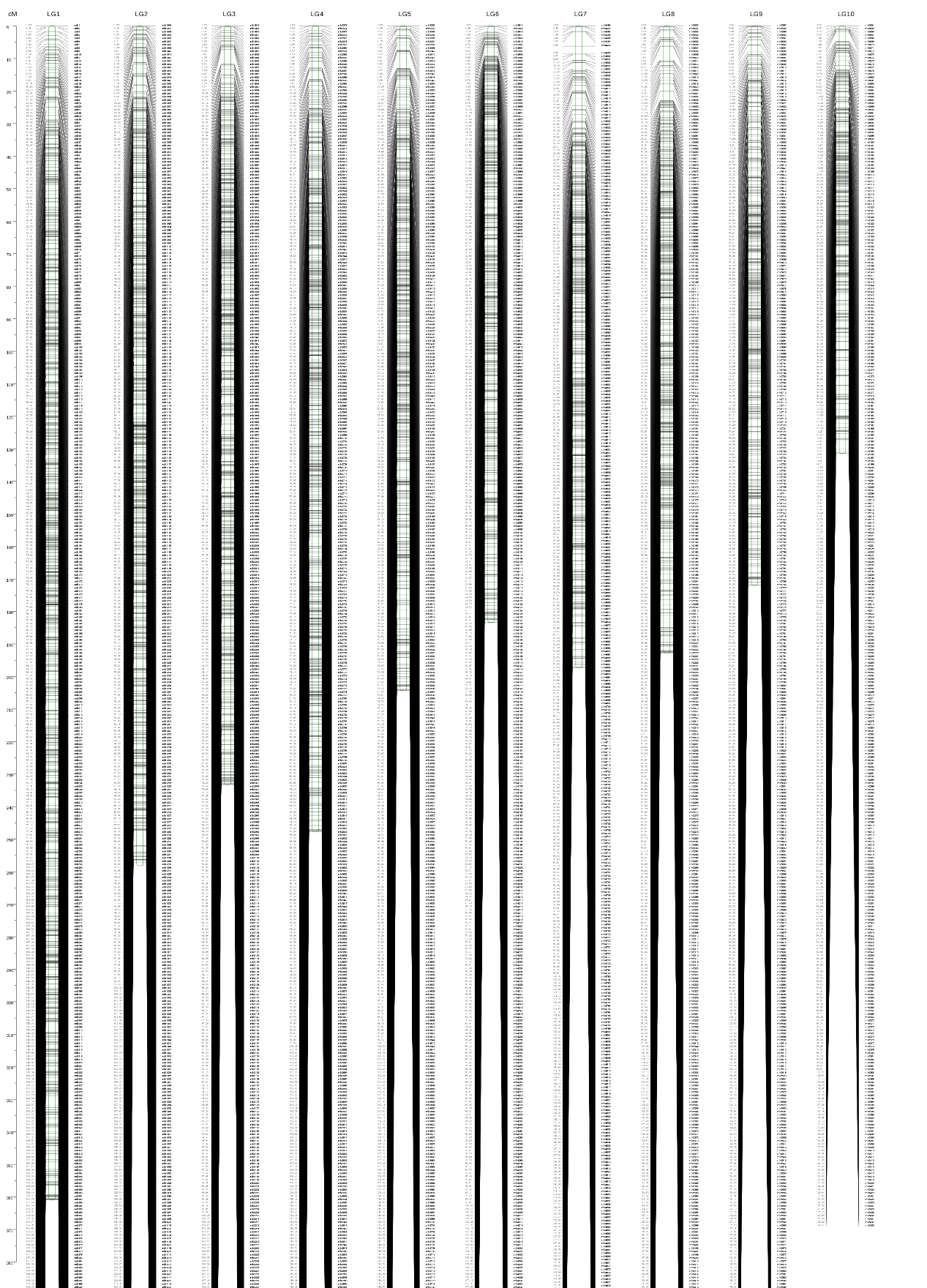
**

**Supplementary Figure 3.** **Comparison of the physical and genetic maps derived from the 6433 bin markers.** The order of the bin markers depends on their physical positions. The genetic map positions are on the left side of each linkage group (LG), and the marker names are along the right side.

**Supplementary Figure 4.** **Bin length distribution.**

**
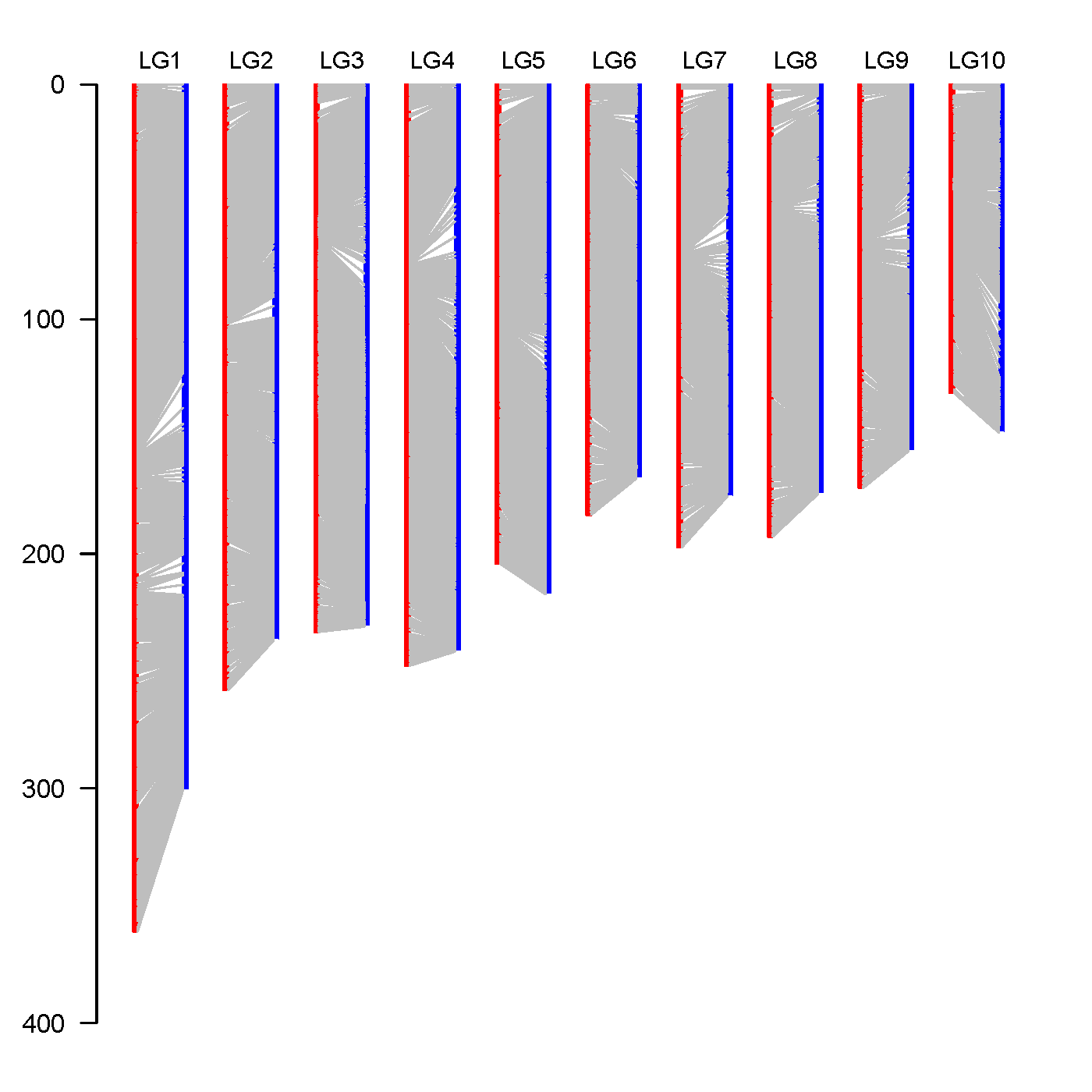
**

**Supplementary Figure 5.** **Collinearity analysis of maize marker linkage groups (LGs) with the maize reference genome B73 RefGen_V4.** Red lines indicate the linear order and genetic distances of the maize marker linkage groups, while the blue lines indicate the linear order of the physical positions in the maize reference genome.


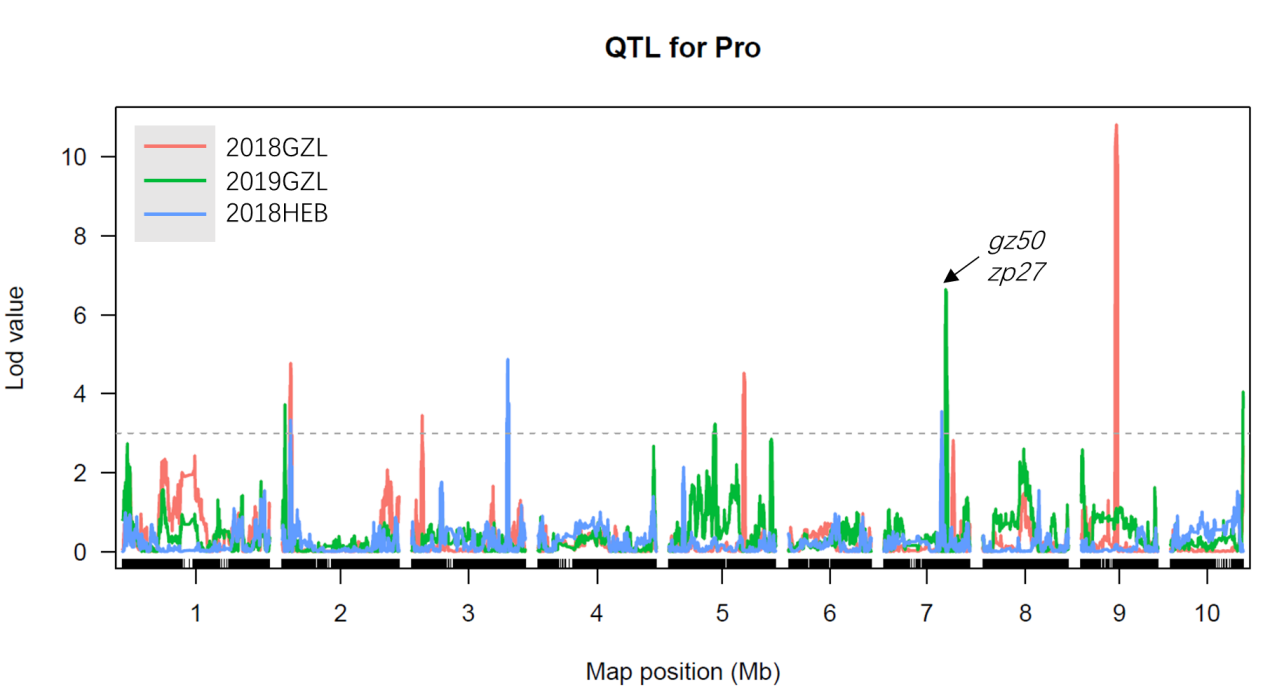


**Supplementary Figure 6. Distribution of PC QTLs across the entire genome in different environments.** 2018GZL, 2019GZL, and 2018HEB represent the environments of Gongzhuling in 2018, Gongzhuling in 2019, and Harbin in 2018, respectively. The curves indicate the physical positions (x-axis) of bin markers against the LOD scores (y-axis) for the QTLs detected on each of the ten chromosomes. Different colors represent different environments. The gray dashed lines represent the LOD threshold.


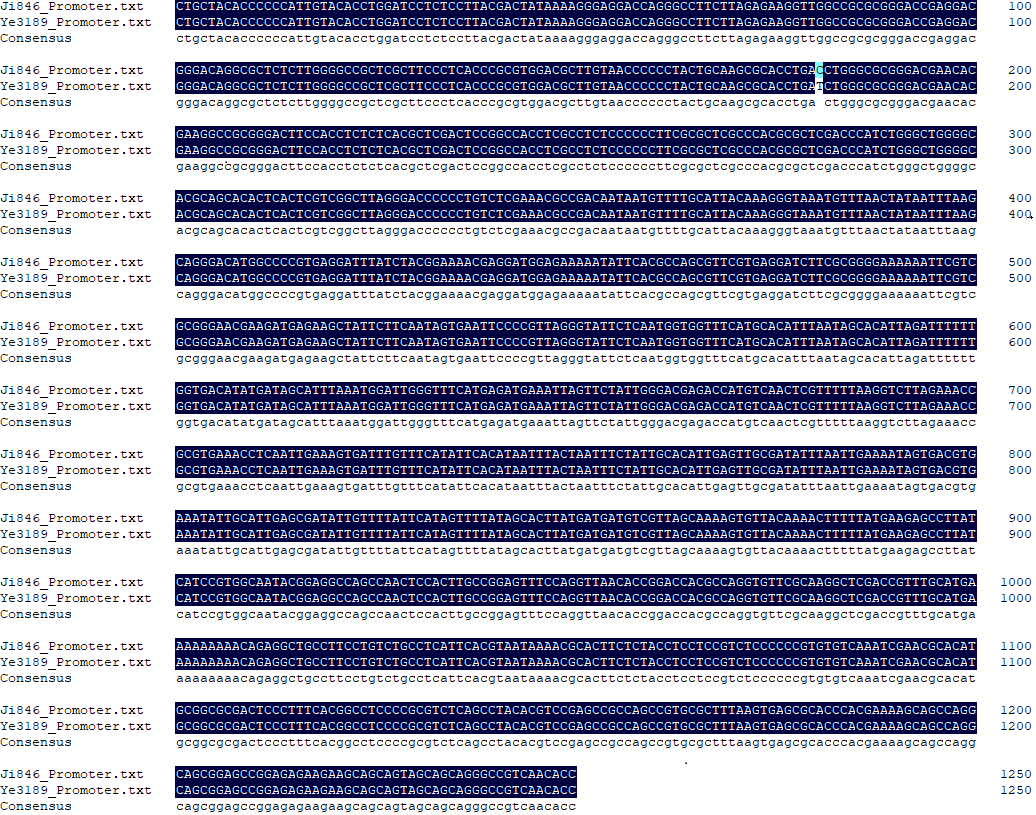


**Supplementary Figure 7. The promoter sequence alignment of *Zm00001d002625* between Ji846 and Ye3189.** The light blue fill indicate the diverse base sites between two sequences. The sequence is located 1250 bp upstream of the initiation codon ATG of *Zm00001d002625.*
